# Me, myself, bye: regional alterations in glutamate and the experience of ego dissolution with psilocybin

**DOI:** 10.1038/s41386-020-0718-8

**Published:** 2020-05-23

**Authors:** N. L. Mason, K. P. C. Kuypers, F. Müller, J. Reckweg, D. H. Y. Tse, S. W. Toennes, N. R. P. W. Hutten, J. F. A. Jansen, P. Stiers, A. Feilding, J. G. Ramaekers

**Affiliations:** 1 Department of Neuropsychology and Psychopharmacology, Faculty of Psychology and Neuroscience, Maastricht University, P.O. Box 616, 6200 MD Maastricht, the Netherlands; 2 Department of Psychiatry (UPK), University of Basel, Basel, Switzerland; 3 Institute of Legal Medicine, University of Frankfurt, Kennedyallee 104, D-60596 Frankfurt/Main, Germany; 4 Department of Radiology and Nuclear Medicine, Maastricht University Medical Center+ (MUMC+), Maastricht, the Netherlands; 5 School for Mental Health and Neuroscience, Maastricht University Medical Center, P. Debyelaan 25, Maastricht, the Netherlands; 6 The Beckley Foundation, Beckley Park, Oxford, OX3 9SY UK

**Keywords:** Consciousness, Neurotransmitters, Human behaviour, Neurophysiology

## Abstract

There is growing interest in the therapeutic utility of psychedelic substances, like psilocybin, for disorders characterized by distortions of the self-experience, like depression. Accumulating preclinical evidence emphasizes the role of the glutamate system in the acute action of the drug on brain and behavior; however this has never been tested in humans. Following a double-blind, placebo-controlled, parallel group design, we utilized an ultra-high field multimodal brain imaging approach and demonstrated that psilocybin (0.17 mg/kg) induced region-dependent alterations in glutamate, which predicted distortions in the subjective experience of one’s self (ego dissolution). Whereas higher levels of medial prefrontal cortical glutamate were associated with negatively experienced ego dissolution, lower levels in hippocampal glutamate were associated with positively experienced ego dissolution. Such findings provide further insights into the underlying neurobiological mechanisms of the psychedelic, as well as the baseline, state. Importantly, they may also provide a neurochemical basis for therapeutic effects as witnessed in ongoing clinical trials.

## Introduction

Psychedelics are a class of psychoactive substances, which induce profoundly altered states of consciousness, including transient and dose-dependent distortions in the subjective experience of one’s self [1]. Termed ego dissolution [2], this phenomenon is characterized by the reduction in the self-referential awareness that defines normal waking consciousness, ultimately disrupting self-world boundaries and increasing feelings of unity with others’ and one’s surroundings [3]. Importantly, there is a renewed interest in the use of these substances in the treatment of various psychiatric conditions characterized by distortions of the self-experience [4, 5]. Recent clinical studies have suggested that these substances can increase well-being [4, 6–11] and provide therapeutic relief for those suffering from anxiety, depression, and addiction [4, 9, 12–15].

Converging evidence suggests that classical psychedelics, such as lysergic acid diethylamide (LSD), psilocybin, and dimethyltryptamine (DMT), stimulate serotonin (5-HT_2A_) receptors located on cortical pyramidal neurons, which is the suggested primary mechanism of action for their hallucinogenic effect [16–22]. Nevertheless, accumulating evidence from preclinical studies also emphasizes the role of the glutamate system in 5-HT_2A_ receptor-mediated effects on brain function [19, 23, 24] and behavior [17]. Specifically, it has been suggested that activation of 5-HT_2A_ receptors leads to a glutamate-dependent increase in activity of pyramidal neurons in the prefrontal cortex [18, 19, 25, 26], subsequently modulating prefrontal network activity [16]. Furthermore, the increase in extracellular glutamate has been suggested to activate AMPA receptors located on the same neurons, increasing expression of brain-derived neurotrophic factor (BDNF) [16, 27, 28], a protein implicated in neuronal survival and growth, and decreased in pathological populations [29]. Taken together, it has been suggested that 5-HT_2A_ receptor-mediated glutamate release is the final common pathway for the acute actions of psychedelics, and a potential underlying mechanism of therapeutic effects [16]. However, no study has investigated the acute effect of a psychedelic on brain glutamate levels in humans, and its relationship with established psychedelic-induced alterations on brain function and behavior.

The present study was designed to establish the contribution of glutamate to the psychedelic state by using ultra-high field (7T) proton magnetic resonance spectroscopy (MRS) that allows in vivo assessment of glutamate in designated brain areas. First, we assessed the acute influence of the classic psychedelic, psilocybin, on glutamate concentration levels in the human brain. Then we assessed the association between glutamate levels, and key features of the psychedelic state, e.g., the experience of ego dissolution, and disrupted resting state network (RSN) functional connectivity (FC). It has been repeatedly found that LSD, DMT, and psilocybin decrease within-network connectivity in several RSNs while increasing connectivity across such networks [30–36]. Affected RSNs include the default mode network (DMN), an interconnected group of brain structures including the medial prefrontal cortex (mPFC), posterior cingulate cortex, and inferior parietal lobule [37, 38]. Importantly, the DMN in particular has become closely associated with self-referential mental activity [37, 39], and psychedelic-induced alterations in DMN function have been repeatedly implicated in the experience of ego-dissolution [33, 40–42].

Relative glutamate concentrations were quantified in the mPFC and the hippocampus. These areas were chosen based on previous anatomical, functional, and behavioral evidence implicating them as potential key regions in modulating the psychedelic experience. Specifically, both areas contain a high density of 5-HT_2A_ receptors [43], which is among the most abundant 5-HT receptor expressed in these regions [44, 45]. Functionally, pre-clinical studies have shown increased glutamate concentrations in the mPFC after 5-HT_2A_ agonism [18, 19, 25, 26], whereas in humans increased glucose, indicative of higher metabolic demands due to increased cell excitability, has been found in frontal and temporal regions after psilocybin [46]. Finally, both areas anatomically overlap with the DMN, with the mPFC recognized as a major hub [37, 38], and a decoupling between the DMN and medial temporal lobe (MTL; especially hippocampal regions) hypothesized to be a key mechanism in the experience of ego dissolution [47–49].

Resting state functional magnetic resonance imaging (rsfMRI) was used to assess RSN FC. Within-network FC assessment followed the approaches of previous studies to allow for comparability [32], and subjective state was characterized via a well-established altered states of consciousness questionnaire [2, 50], and a validated questionnaire to assess ego dissolution [1]. As MRS captures a range of brain metabolites, we also performed an exploratory analysis to assess whether psilocybin affected other metabolites of interest, including γ-aminobutyric acid (GABA) [17], and markers of neuronal integrity and glial activation, including n-acetyl-aspartate (NAA), and myo-inositol (mI).

## Materials and methods

A detailed description of the experimental procedure, image acquisition, MRS quantification, and rsfMRI analysis is provided in the Supplementary methods, and briefly summarized here.

The present study employed a randomized, placebo-controlled, double-blind, parallel group design. Sixty healthy participants, with previous experience with a psychedelic drug but not within the past 3 months, were allocated to a treatment condition (0.17 mg/kg psilocybin or placebo, p.o.). Groups were matched for age, sex, and education level.

This study was conducted according to the code of ethics on human experimentation established by the declaration of Helsinki (1964) and amended in Fortaleza (Brazil, October 2013) and in accordance with the Medical Research Involving Human Subjects Act (WMO) and was approved by the Academic Hospital and University’s Medical Ethics committee. All participants were fully informed of all procedures, possible adverse reactions, legal rights and responsibilities, expected benefits, and their right for voluntary termination without consequences.

### Image acquisition

Participants underwent structural MRI (50 min post treatment), single-voxel proton MRS in the mPFC (65 min post) and hippocampus (95 min post), and fMRI (102 min post), during peak subjective drug effects. Images were acquired on a MAGNETOM 7T MR scanner.

Spectroscopic voxels were placed by a trained operator at the mPFC (voxel size = 25 × 20 × 17 mm^3^) and the right hippocampus (voxel size = 37 × 15 × 15 mm^3^). Spectra were acquired with the stimulated echo acquisition mode [51] sequence (TE = 6.0 ms, TR = 5.0 s, 64 averages). Outcome measures for MRS were concentration ratios of glutamate, GABA, NAA, and mI, to total Creatine (tCr, Creatine + Phospho-Creatine), because using tCr as the internal reference inherently corrects for variabilities caused by transmit or receive RF inhomogeneity, magnetic field drift, and CSF inclusion in the voxel [52].

In addition, 258 whole-brain EPI volumes were acquired at rest (TR = 1400 ms; TE = 21 ms; field of view=198 mm; flip angle = 60°; oblique acquisition orientation; interleaved slice acquisition; 72 slices; slice thickness = 1.5 mm; voxel size = 1.5 × 1.5 × 1.5 mm). During scanning, participants were shown a black cross on a white background, and instructed to focus on the cross while clearing their mind and laying as still as possible.

### Processing of imaging data

Spectroscopy data was analyzed with LCModel version 6.3–1H.

Resting state data was processed and analyzed using the CONN toolbox 18.b [53]. All volumes were realigned, unwarped, segmented into gray and white matter and cerebrospinal fluid, normalized into a standard stereotactic space (Montreal Neurological Institute), and smoothed with a 6 mm full width at half maximum Gaussian kernel.

Independent component analysis (ICA) was performed using group-ICA procedures implemented in the CONN toolbox following previously described methods [54]. Independent components were restricted to 20 in order to allow comparisons with 10 established RSNs [55] and previous studies on psilocybin [42] and LSD [32, 33].

### Subjective state

The 5 Dimensions of Altered States of Consciousness (5D-ASC) scale [50] and the Ego Dissolution Inventory (EDI) [1] were administered 360 min after drug administration, as retrospective measures of drug effects.

### Pharmacokinetic measures

Venous blood samples were collected after treatment administration (at 80, 150, and 360 min) in order to assess concentrations of psilocin, the main metabolite of psilocybin.

### Statistical analysis

Statistical analysis of metabolite concentration levels and questionnaire responses were conducted in IBM SPSS Statistics 24 using nonparametric Mann–Whitney *U* tests.

For the assessment of within-network FC, the unthresholded, binarized ICA component images were compared between placebo and drug conditions (two-sample *t*-test). Parametric statistics were used (voxel threshold *p* < 0.001 uncorrected, cluster threshold *p* < 0.05 cluster-size, false discovery rate (FDR) corrected, two-sided).

For the assessment of between-network FC, unthresholded, binarized maps of RSNs obtained from the ICA analysis were imported as ROIs and the weighted sums of the time series were extracted. Time courses between all RSNs were then compared for both conditions using bivariate correlations. The resulting correlation coefficients were compared between placebo and drug conditions (two-sample *t*-test). Results were corrected for multiple comparisons using FDR.

Canonical correlations [56] were conducted to evaluate the association between psilocybin-induced changes in (i) relative glutamate concentration levels in the mPFC and hippocampus, (ii) ratings of ego dissolution, including two dimensions of the 5D-ASC (oceanic boundlessness and anxious ego dissolution) and scores on the EDI, and (iii) within-network resting state FC, using extracted connectivity strength (beta) values. Variables were separated into two sets; set 1 included biological variables as predictors [(i) and (iii)] and set 2 included the subjective variables as criterion (ii*)*. An iterative imputation approach was performed to fill in missing data points, when applicable.

The alpha criterion of significance of all tests was assumed at *p* < 0.05.

## Results

### Demographic variables and psilocin concentration levels

The psilocybin group (*n* = 30) and the placebo group (*n* = 30) did not differ in respect to demographic variables (Table S1).

Mean (S.E.) concentrations of psilocin in serum are given in Table S2. Concentrations reached a peak 80 min postdrug administration (15.61 ± 1.66 ng/mL), and then began to fall (360 min post 4.85 ± 0.54 ng/mL). The measured concentrations are in accordance with the applied oral dose [57].

### Acute effect of psilocybin on subjective state

**Fig. 1:**
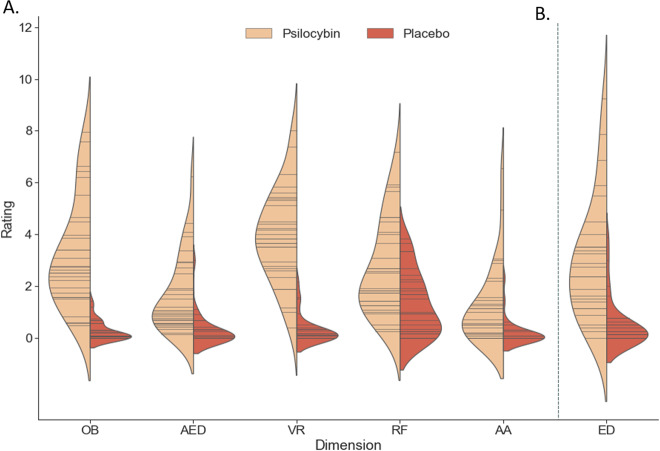
Retrospective ratings of drug effects. Violin plots displaying reported scores on the 5 main dimensions of the 5D-ASC (**a**), ratings on the ego dissolution inventory (**b**) for each treatment group. Each stick in the violin indicates a data point, whereas the density is scaled to the relative count across all bins.

Administration of psilocybin was associated with increased ratings on all (sub)dimensions of the 5D-ASC (*U* = 13.5–225; *p* ≤ 0.001 effect size = 0.43–0.84, Fig. 1a; S1), and on the EDI (*U* = 91.5, *p* < 0.001, effect size = 0.67, Fig. 1b).

### MRS results

Spectral quality for each treatment condition is reported in Table 1. Overview of data points that did not meet the data quality criteria check can be found in Table S6.

**Table 1:** Mean (SD) spectral quality per group. Only data points with an SNR > 10, FWHM < 0.1, and a CRLB < 20% were included in the final analysis.

Parameter	Psilocybin	Placebo	*t* value	*P* value
**Medial prefrontal cortex**				
Relative Cramer–Rao lower bound (%); *n*				
* Glutamate*	2.62 (0.57); 24	2.71 (0.60); 28	−0.54	0.58
* GABA*	13.33 (3.52); 15	14.65 (3.33); 17	−1.08	0.29
* NAA* + *NAAG*	2.37 (0.49); 24	2.32 (0.55); 28	0.36	0.71
* Myoinositol*	4.33 (2.14); 24	4.11 (1.42); 28	0.45	0.65
Signal to noise ratio	36.87 (4.77)	37.96 (6.52)	−0.67	0.50
Full-width at half-maximum peak height	0.04 (0.01)	0.04 (0.01)	−0.307	0.76
**Hippocampus**				
Relative Cramer–Rao lower bound (%); *n*				
* Glutamate*	5.85 (2.32); 21	5.00 (1.73); 25	1.43	0.16
* GABA*	16.60 (1.67); 5	14.50 (3.07); 8	1.39	0.19
* NAA* + *NAAG*	2.90 (0.77); 21	2.96 (0.93); 25	−0.22	0.83
* Myoinositol*	4.70 (1.26); 20	4.62 (3.12); 24	0.10	0.92
Signal to noise ratio	20.57 (5.61)	21.16 (6.86)	−0.31	0.75
Full-width at half-maximum peak height	0.07 (0.01)	0.06 (0.01)	1.80	0.08

*N* refers to the number of data points that met the criteria, per metabolite, per group, and were included in the analysis.

#### Medial prefrontal cortex

**Fig. 2:**
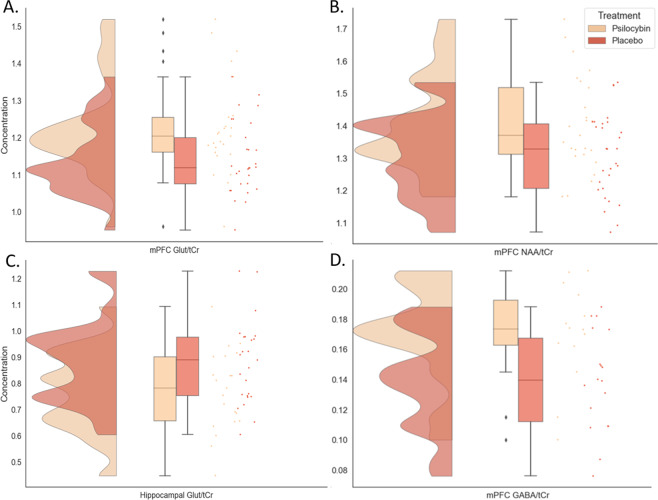
Raincloud plots displaying metabolite concentrations in the mPFC and the hippocampus, which demonstrated significant differences between treatment groups. **a** glutamate in the mPFC, **b** NAA in the mPFC, **c** glutamate in the hippocampus, **d** GABA in the mPFC. The plot consists of a probability density plot, a boxplot, and raw data points. In the boxplot, the line dividing the box represents the median of the data, the ends represent the upper/lower quartiles, and the extreme lines represent the highest and lowest values excluding outliers. The code for raincloud plot visualization has been adapted from Allen et al. [99].

As hypothesized, glutamate/total creatine (glutamate) in the mPFC was higher after psilocybin, compared with placebo (mean ± S.E.; psilocybin: 1.23 ± 0.02; placebo: 1.14 ± 0.02, *U* = 200.50, *p* = 0.01, effect size = 0.80, Fig. 2a).

In addition, tNAA/total creatine (psilocybin: 1.41 ± 0.03; placebo: 1.31 ± 0.02, *U* = 210.0, *p* = 0.02, effect size = 0.72, Fig. 2b), and GABA/total creatine (psilocybin: 0.17 ± 0.01; placebo: 0.14 ± 0.01, *U* = 66.0, *p* = 0.01, effect size = 0.99, Fig. 2d) were higher after psilocybin, compared with placebo.

#### Hippocampus

In contrast, glutamate in the hippocampus (psilocybin: 0.77 ± 0.03; placebo: 0.88 ± 0.03, *U* = 163.50, *p* = 0.03, effect size = 0.69, Fig. 2c) was lower after psilocybin, compared to placebo.

No other significant differences were seen between groups in regards to relative concentrations of GABA, tNAA, mI, or total creatine concentration. See Table S3 for means of all investigated metabolite concentration, additional metabolites acquired in the spectra, and Figure S2 for representative spectra and voxel placement.

### Resting state networks

After quality control, the final sample consisted of 22 participants in the psilocybin group and 26 in the placebo group. There were no significant differences between groups in regards to head motion parameters. See supplementary for exclusion criteria and assessed differences between groups.

#### Independent component analysis

There was a good agreement between most of the components identified in our analysis and the templates provided by Smith et al. [55]. We were able to identify the visual networks 1–3 (*r* = 0.80, *r* = 0.73 and *r* = 0.64, respectively), the cerebellar network (*r* = 0.38), the auditory network (*r* = 0.43), the executive control network (*r* = 0.58) and the frontoparietal networks 1 (*r* = 0.50) and 2 (*r* = 0.47). In contrast, we were not able to assign a single component to the DMN and the sensorimotor network, as these networks were split up in sub-components, as already observed in multiple studies [58–60]. The DMN consisted of two components (anterior DMN: *r* = 0.34 and posterior DMN: *r* = 0.52) and the sensorimotor network consisted of three components (somatosensory network: *r* = 0.40, lateral motor network: *r* = 0.32, medial motor network: *r* = 0.24). In order to allow a comprehensive exploration, we decided to include all of these components in further analysis. For this purpose, the respective sub-components were labeled according to common terminology [60] but in deviation from previous work [55] (i.e., the components were labeled as anterior and posterior DMN, medial and lateral motor network, and somatosensory network). The remaining seven components reflected noise or networks that were not relevant for this analysis.

#### Within-network connectivity

**Fig. 3:**
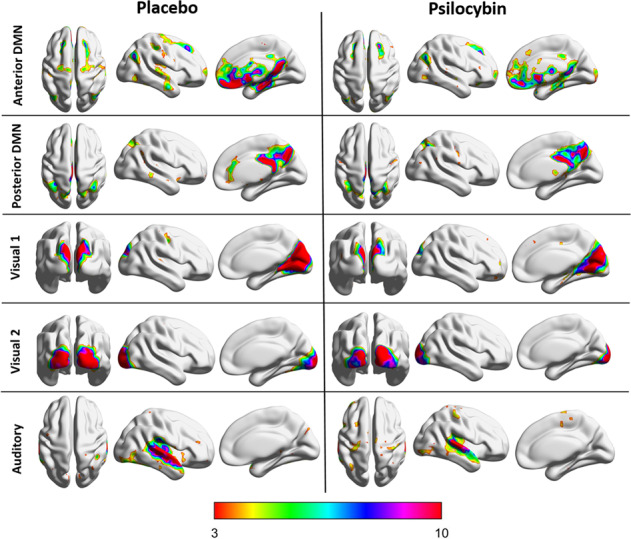
Within-network resting state functional connectivity. Resting state networks that demonstrated significant differences in within-network functional connectivity, for each group (placebo and psilocybin).

Within the respective network, significantly less coactivation under the drug condition relative to placebo was found in visual network 1 and 2, both subcomponents of the DMN (anterior and posterior), and the auditory network (Fig. 3; Table S4).

#### Between-network connectivity

Widespread increases in between-network FC were observed under psilocybin compared to placebo. Except the lateral motor network, all investigated networks were affected to some extent (Table S5).

### Relationship between psilocybin-induced changes in brain and behavior

A canonical correlation analysis was conducted using the four biological variables as predictors of the three ego dissolution variables, to evaluate the multivariate shared relationship between the two variable sets. The analysis yielded three functions with squared canonical correlations (*R*_*c*_^2^) of 0.363, 0.282, and 0.253 for each successive function. The full model across all functions was statistically significant *F*(12,61.14) = 2.47, *p* = 0.008), explaining 65.9% of the variance.

Given the *R*_*c*_^2^ effects for each function, the first two functions were significant (*p* = 0.008 and *p* = 0.016, respectively) and considered noteworthy in the context of this study, with function 1 explaining 36.3% of the variance, and function 2 explaining 28.2% of the variance.

Table 2 presents the standardized canonical function coefficients, the structure coefficients (*r*_s)_, and the squared structure coefficients (*r*_*s*_^2^*)* for functions 1 and 2, as well as the communalities (*h*^2^) across the two functions for each variable. Function 1 indicated that the dominant contributor was anxious ego dissolution (AED), with oceanic boundlessness (OB) making a secondary contribution. In regards to predictors, mPFC glutamate was the dominant predictor, with anterior DMN FC making a secondary contributions. These results suggest that the strongest predictor of negatively experienced ego dissolution (i.e., AED) was the increase in mPFC glutamate.

**Table 2:** Canonical solution for biological variables predicting ego dissolution for Functions 1 and 2. *r*_s_ > |0.45| and *h*^2^ > 45% are underlined and deemed valuable contributors.

Variable	Function 1			Function 2			
	Coef	*r*_s_	*r*_s_^2^ (%)	Coef	*r*_s_	*r*_s_^*2*^ *(%)*	*h*^2^(%)
Oceanic boundlessness	−1.439	−0.568	32.2	0.322	−0.689	47.5	79.7
Anxious ego dissolution	−0.810	−0.681	46.4	0.727	−0.365	13.3	59.7
Ego dissolution inventory	1.347	−0.274	7.5	−1.389	−0.956	91.4	98.9
*R*_*c*_^2^			36.3			28.2	
Glutamate/tCr hippocampus	−0.648	0.113	1.3	1.102	0.990	98.0	99.3
Glutamate/tCr mPFC	−0.724	−0.630	40.0	0.036	−0.047	0.2	40.2
Anterior DMN	0.853	0.520	27.0	−0.166	0.400	16.0	43.0
Posterior DMN	0.551	0.313	9.7	−0.047	0.484	23.4	33.1

*Coef* standardized canonical function coefficients, *r*_*s*_ structure coefficients, *r*_*s*_^*2*^ squared structure coefficient, *h*^*2*^ communality coefficient, *R*_*c*_^*2*^ squared canonical coefficient.

Function 2 indicated that the dominant contributor was ratings on the EDI, with OB as a secondary contribution. As for the predictors, hippocampal glutamate was the strongest predictor, with posterior DMN FC making a secondary contribution. These results suggest that the strongest predictor of positively experienced ego dissolution was the decrease in hippocampal glutamate.

## Discussion

The present study demonstrates the first attempt to assess the acute effects of psilocybin on glutamate levels in key areas of the human brain, which may play a major role in the actions of serotonergic psychedelics. Using an ultra-high field multimodal MRI approach, we demonstrated that, compared with placebo, psilocybin-induced region-dependent alterations in neurometabolite concentrations. Specifically, participants who received psilocybin demonstrated higher relative glutamate concentration levels in the mPFC, and lower relative glutamate concentration levels in the hippocampus. Analyses indicated that region-dependent alterations in glutamate were also correlated with different dimensions of ego dissolution. Whereas changes in mPFC glutamate were found to be the strongest predictor of negatively experienced ego dissolution, changes in hippocampal glutamate were found to be the strongest predictor of positively experienced ego dissolution.

Previous studies have demonstrated that the mPFC is highly enriched with 5-HT_2A_ receptors located primarily on layer V pyramidal neurons [61], and modulate excitatory transmission in cortical circuits [43, 62, 63]. Preclinical studies have demonstrated that activation of such receptors via serotonergic psychedelics results in a predominantly excitatory response [18, 64] via an increase in glutamate release, as observed in humans for the first time in this study. A glutamatergic increase in this area is also in accordance with human functional imaging studies which have demonstrated a hyperfrontal regional cerebral blood flow (CBF) pattern after psilocybin [46, 65], and similar 5-HT_2A_ agonist psychedelics [66, 67]. However, we also found that psilocybin administration was associated with higher levels of GABA in this area, results in line with findings that 5-HT_2A_ receptors are also located on GABAergic interneurons [17, 68]. Taken together, findings suggest that activation of 5-HT_2A_ receptors in the mPFC results in both excitation and inhibition of cortical pyramidal cells [17], potentially resulting in an increased metabolic rate in this area, but not necessarily increased neural input or output.

In contrast to the mPFC, the present study demonstrated that participants who received psilocybin demonstrated *lower* relative glutamate concentrations in the hippocampus, suggesting that psilocybin decreases glutamate in this area. Such a decrease is in line with data from a recent functional imaging study with psilocybin, demonstrating reduced absolute CBF in the hippocampus compared with placebo [69], of which the authors proposed two potential mechanisms. Namely, decrements could be due to agonism of 5-HT_2A_ receptors located on GABAergic interneurons [44], which can indirectly inhibit pyramidal neurons, decreasing activation in this area. Conversely, it has also been established that, along with the 5-HT_2A_ receptor, psilocin also has a high affinity for the 5-HT_1A_ receptor [70, 71]. Referred to as serotonin’s principal inhibitory receptor [72], the 5-HT_1A_ receptors highest density is found in the limbic regions of the brain such as the hippocampus [73] where it is expressed on neurons that are postsynaptic to the serotonergic input. Thus lower levels in glutamate as seen in this study, as well as regional decreases reflected in others [69], could be due to activation of postsynaptic inhibitory 5-HT_1A_ receptors. Nevertheless, due to methodological limitations, this study is not able to delineate which mechanism is contributing to the lower levels in glutamate. Further information could have been potentially gained from quantification of GABA in the hippocampus, however we were unable to reliably do so, due to inherent quantification challenges when assessing GABA levels, arising from low brain concentration levels, metabolite signal overlap, and low signal-to-noise in the hippocampus [74, 75]. Future studies with sequences developed to specifically quantify GABA in low signal-to-noise areas should make further attempts to do so, given recent research implicating hippocampal GABA in the pathology of disorders that psychedelics are being investigated to treat [76].

In the current study, psilocybin induced previously established key features of a psychedelic experience: increases in feelings of ego dissolution, and disrupted RSN activity. Psilocybin increased scores on all dimensions of the 5D-ASC [16], as well as on the EDI [1]. In addition, psilocybin altered within-network FC similarly as has been shown with LSD, including decrements in coactivation within the DMN, visual network 1, and the auditory network [32, 33]. Finally, we demonstrated higher between-network FC across all networks, which is similar with previous studies assessing the same after psilocybin [35, 42] and LSD [32, 33].

Finally, we assessed the relationship between psilocybin-induced changes in the brain, and the subjective experience of sense of self. Canonical correlations were conducted to predict increases in ratings of AED, the dimension encompassing the loss of autonomy and self-control of thought processes, intentionality, decision making, and spontaneous movements [46]. Our data support the conclusion that increasing levels of mPFC glutamate were the strongest predictor in regards to feelings of AED, with decreasing anterior DMN FC and hippocampal glutamate being secondary predictors. These findings are in line with previous work, implicating increased frontal metabolism in feelings of AED after psilocybin [46] and ego pathology in the ketamine model of psychosis [77]. Interestingly, AED-associated changes in mood include paranoia, heightened arousal and attention to the surroundings, and anxiety [46]. A paradoxical effect of serotonergic psychedelics is that acutely they have been found to increase feelings of anxiety [6, 78], whereas clinical trials with psychedelic drugs suggest long-term anxiety relief in patients [11, 12, 14]. Accordingly, there is a wide range of animal and human pharmacological evidence supporting the role of the glutamatergic system in anxiety [79], with increases in glutamate in the frontal cortex associated with high versus low state-trait anxiety [80], and reductions corresponding to anxiety-related symptomatic relief [81]. Taken together, the finding that mPFC glutamate was by far the strongest predictor of increased feelings of anxiety, one could propose that acute psychedelic-induced anxiety may be due to localized glutamate-induced hyperfrontality, whereas long-term reductions could be due to agonist-induced 5-HT_2A_ receptor downregulation in this area [72, 82]. Nonetheless, future studies should assess long-term changes in 5-HT_2A_ receptor function in the mPFC, and their relation with subjective effects.

We also assessed the relationship between psilocybin-induced brain changes and feelings of positively experienced ego dissolution, including ratings on the EDI, and scores of OB on the 5D-ASC. We found that the primary predictor of positively experienced ego dissolution was a decrement in hippocampal glutamate, with secondary contributions of mPFC glutamate and posterior DMN integrity. Previous work has implicated both the MTL (containing the hippocampus) and DMN circuitry in the neural correlates of the self [49]. Namely, abnormal function of MTL regions have been implicated in psychotic states [83, 84] and feelings of depersonalization [85] and ego-disturbances [86]. Similarly, studies of drug-induced ego dissolution have found that the decoupling of MTL regions such as the parahippocampus and the DMN correlate positively with feelings of ego dissolution [49, 87], with this decoupling being hypothesized to be one of the main underlying mechanisms of the subjective experience [47–49]. In regards to why this gives rise to ego dissolution, it has been suggested that psychedelic drug-induced decoupling of these regions results in a temporary loss of access of semantic autobiographical information, resulting in a breakdown of one’s personal identity [87]. Our data add to this hypothesis, suggesting that modulations of hippocampal glutamate in particular may be a key mediator in the decoupling underlying feelings of (positive) ego dissolution. Interestingly, although the DMN has been the most implicated RSN in this process, Lebedev et al. [49] found that increases in ego dissolution correlated with decreased FC between the parahippocampal formation and other major networks, such as the salience, frontoparietal, and sensorimotor network; suggesting a key role in this area in particular, as our data also demonstrate. However future research should further assess the contribution of other areas to this experience, such as the posterior cingulate cortex.

Implications of these findings also extend far beyond understanding the neurobiology of the acute psychedelic experience and drug-induced ego dissolution. There is growing evidence that psychedelics can provide therapeutic relief for individuals suffering from increasingly common and difficult to treat disorders such as depression, anxiety, addiction, and post-traumatic stress disorders [4, 9, 11, 88, 89]. Thus understanding the mechanisms by which psychedelics provide symptomatic relief may identify novel therapeutic targets. Interestingly, the degree of ego dissolution has been found to correlate with long-term clinical outcomes [90] and increases in well-being [10, 91]. In addition, a hypothetical (neurobiological) model has been proposed to explain the long-term effects witnessed in clinical trials. It has been suggested that indirect activation of glutamate networks via 5-HT_2A_ receptor agonism increases BDNF, and ultimately enhances neuroplasticity [16]. In line with this, it has been shown in preclinical models that psychedelics increase functional and structural neuroplasticity [92], however evidence in humans is limited, due to restrictions of methodological techniques. Our data provide indirect evidence that psychedelics might have the potential to increase neuroplasticity in the human cortex via increased glutamatergic activity, but not in the hippocampus; findings that are in accordance with previous 5-HT_2A_ receptor activation studies [27, 93, 94]. In addition, psilocybin administration was associated with higher levels of mPFC NAA, a compound regarded as a measure of neuronal viability and function, and decreased in disorders associated with regional neuronal loss and disrupted neuronal function [95].

Of note, compared to previous psychedelic studies, the dose administered was low to moderate [96], and thus not high enough to induce total ego dissolution. However, the aim of this study was not to assess maximal effects of psilocybin, but rather an effective dose that would induce a relevant psychedelic state that participants could endure in the MRI scanner. Our data demonstrate that the dose was effective, inducing significantly higher levels of both positively and negatively experienced ego dissolution compared with placebo, as well as the other subjective effects representative of a psychedelic state (Figs. 1, S1). Furthermore, although BOLD sensitivity is increased by the use of ultra-high magnetic fields, geometric distortions become more prominent, which could have affected our BOLD signal in inferior brain regions [97], and our scan time was arguably short from a test–retest reliability standpoint [98]. Nevertheless, our results are similar to aforementioned studies [32, 33, 35, 42] who acquired their data at a lower field strength, with varying scanning lengths. Finally, an inherent difficulty of studying substances with such salient subjective effects is maintaining the treatment blind. Thus, it could be suggested that participant recognition of the treatment condition could affect neural and subjective results, emphasizing the importance of active placebo conditions or cross-psychotropic comparisons in future trials.

In conclusion, our data demonstrate that the serotonergic psychedelic, psilocybin, acutely induces region dependent alterations in glutamate that correlate with established behavioral changes during the psychedelic state. Such findings provide further insights into the underlying neurobiological mechanisms of the psychedelic state, and importantly, provide a neurochemical basis for how these substances alter individuals’ sense of self, and may be giving rise to therapeutic effects witnessed in ongoing clinical trials.

## Funding and disclosure

This study is part of the Beckley/Maastricht Research Programme. The Beckley Foundation made a financial contribution to the study. The authors report no other relevant funding, and all authors report no potential conflicts of interest.

## Supplementary information

Supplemental information
