# Supplementary material for: Me, myself, bye: regional alterations in glutamate and the experience of ego dissolution with psilocybin
Source: Neuropsychopharmacology. 2020 May 23;45(12):2003–11. doi: 10.1038/s41386-020-0718-8 (PMC7547711; doi:10.1038/s41386-020-0718-8)
Supplement: Supplementary file 1 — Supplemental information [file 41386_2020_718_MOESM1_ESM.docx]

**Supplementary Information for**

Me, myself, bye: regional alterations in glutamate and the experience of ego dissolution with psilocybin.

***Corresponding author**

Email: natasha.mason@maastrichtuniversity.nl

**Manuscript details**

Word count text: 2419

Display Items (Figures and Tables): 8

Figure S1. Violin plots displaying reported scores on the 11 sub-dimensions of the 5D-ASC.


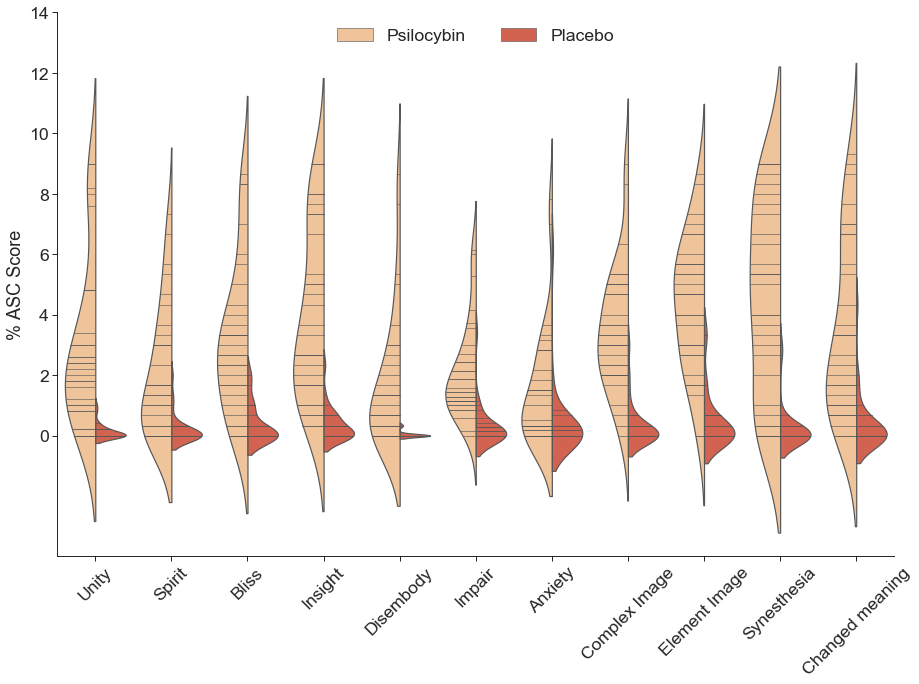


Figure S2. Example LC-Model fitted 1H-MRS data for the medial prefrontal cortex (A) and hippocampus (B). The black line spectra corresponds to the phased 1H-MRS data with the LC-Model fits overlaid (red). The residual spectra (raw data minus the LC-Model fit) are displayed below the spectrum.


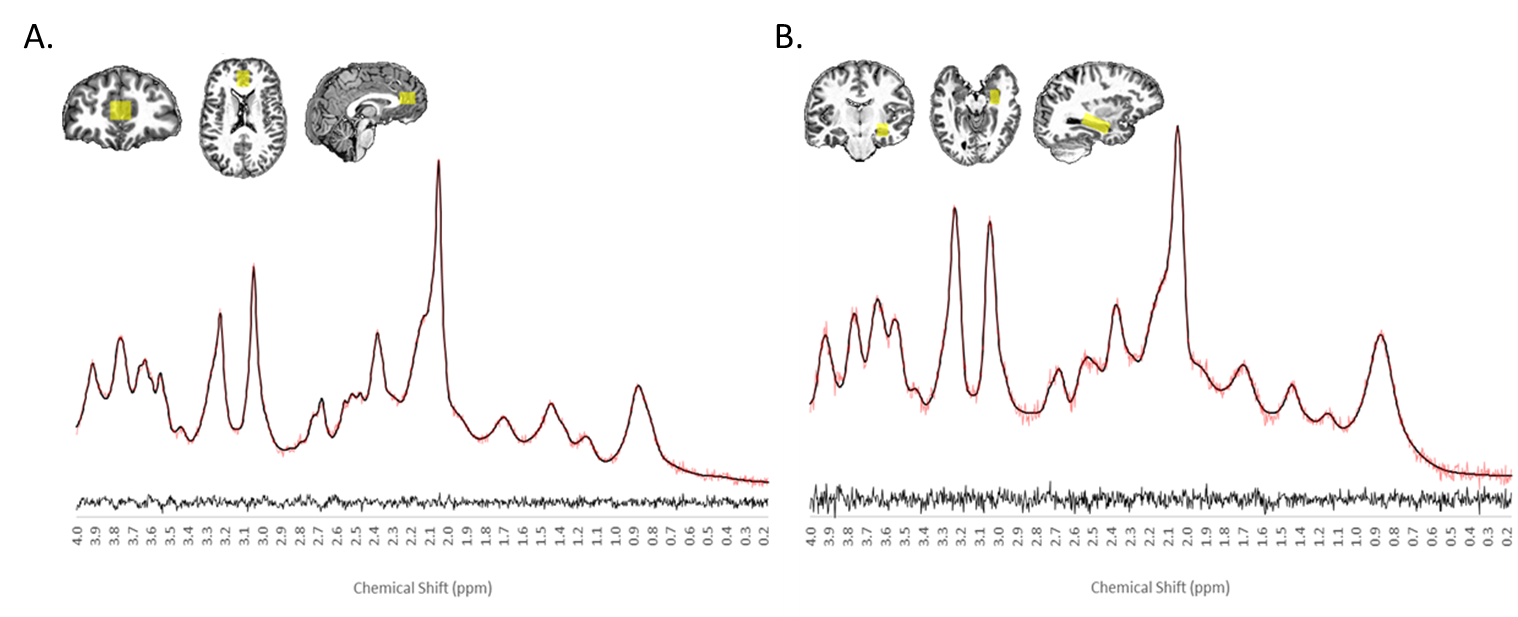


| Table S1. Mean subject characteristics (SD) and history of drug use for healthy participants in the psilocybin and the placebo condition (N=60). | | | | |
| --- | --- | --- | --- | --- |
| Variable | Psilocybin | Placebo | Value | *P* value |
| Gender (male/female), n, total | 18/12, 30 | 17/13, 30 | χ2 =0.07 ^‡^ | 0.79 |
| Age, years | 22.73 (2.90) | 23.20 (3.65) | *t*=-0.55^†^ | 0.60 |
| History of psychedelic use, years | 2.92 (2.62) | 2.19 (2.55) | *t*=1.04 | 0.30 |
| Lifetime psychedelic use, number of occasions | 9.53 (16.81) | 5.47 (8.24) | *t*=1.09 | 0.28 |
| Cannabis consumption, per month | 2.67 (3.14) | 3.24 (4.91) | *t*=-0.47^†^ | 0.64 |
| Alcohol consumption, glasses per week | 5.47 (4.78) | 5.82 (4.02) | *t*=0.31^†^ | 0.76 |
| Caffeine consumption, glasses per week | 10.00 (7.75) | 9.38 (8.10) | *t*=0.30^†^ | 0.76 |
| Nicotine consumption, cigarettes per week | 3.82 (15.66) | 7.67 (15.49) | *t*=-0.96^†^ | 0.34 |
| *Significant *P* values  ^†^Independent *t* test  ^‡^χ2 test for frequency data | | | | |

| Table S2. Time course for mean (S.E.) concentrations of psilocin in serum (ng/ml), as determined by liquid chromatography-mass spectrometry (LC-MSMS). | |
| --- | --- |
| Time relative to treatment intake (minutes) | Psilocin (ng/ml) |
| 80 | 15.61 (1.66) |
| 150 | 12.86 (1.13) |
| 360 | 4.85 (0.54) |

| Table S3. Mean (S.E.) metabolite concentrations for each treatment condition, including metabolites that were quantified in the spectra, but not of focus for this study. Also included are grey matter, white matter, and CSF amounts in each voxel. **p* < .05 | | | | | | | | | | | | | | | | |  |  |  |  |  |  |  |
| --- | --- | --- | --- | --- | --- | --- | --- | --- | --- | --- | --- | --- | --- | --- | --- | --- | --- | --- | --- | --- | --- | --- | --- |
| **Focus of study** | Glu/tCr | | | GABA/tCr | |  | NAA + NAAG/tCr | |  | mI/tCr | |  | tCr | | | |  |  |  |  |  |  |  |
|  | Psilocybin | Placebo | *p* | Psilocybin | Placebo | *p* | Psilocybin | Placebo | *p* | Psilocybin | Placebo | *p* | Psilocybin | Placebo | *p* |  |  |  |  |  |  |  |  |
| Medial prefrontal cortex | 1.23 (0.02) | 1.14 (0.02) | .01* | 0.17 (0.01) | 0.14 (0.01) | .01* | 1.41 (0.03) | 1.31 (0.02) | .02* | 0.78 (0.03) | 0.78 (0.02) | .95 | 3.69 (0.10) | 4.10 (0.16) | .07 |  |  |  |  |  |  |  |  |
| Hippocampus | 0.77 (0.03) | 0.88 (0.03) | .03* | 0.17 (0.01) | 0.20 (0.04) | .88 | 1.41 (0.05) | 1.36 (0.04) | .40 | 1.00 (0.05) | 1.04 (0.04) | .39 | 4.28 (0.21) | 4.20 (0.19) | .43 |  |  |  |  |  |  |  |  |
| **Extra** | Gln/tCr | | | Glx/tCr | | | Cr/tCr | | | PCr/tCr | | | NAA/tCr |  |  |  |  |  |  |  |  |  |  |
|  | Psilocybin | Placebo | *p* | Psilocybin | Placebo | *p* | Psilocybin | Placebo | *p* | Psilocybin | Placebo | *p* | Psilocybin | Placebo | *p* |  |  |  |  |  |  |  |  |
| Medial prefrontal cortex | 0.28 (0.01) | 0.28 (0.01) | .71 | 1.51 (0.03) | 1.42 (0.02) | .03* | 0.51 (0.04) | 0.46 (0.33) | .47 | 0.63 (0.03) | 0.64 (0.03) | .78 | 1.35 (0.03) | 1.25 (0.02) | .03* |  |  |  |  |  |  |  |  |
| Hippocampus | 0.21 (0.03) | 0.18 (0.02) | .56 | 0.87 (0.05) | 1.02 (0.03) | .01* | 0.72 (0.07) | 0.69 (0.06) | .68 | 0.79 (0.05) | 0.73 (0.05) | .36 | 1.41 (0.05) | 1.36 (0.04) | .40 |  |  |  |  |  |  |  |  |
| **Extra** | tCho/tCr | | | GSH/tCr | | | Grey matter | | | White matter | | | CSF |  |  |  |  |  |  |  |  |  |  |
|  | Psilocybin | Placebo | *p* | Psilocybin | Placebo | *p* | Psilocybin | Placebo | *p* | Psilocybin | Placebo | *p* | Psilocybin | Placebo | *p* |  |  |  |  |  |  |  |  |
| Medial prefrontal cortex | 0.16 (0.01) | 0.15 (0.01) | .73 | 0.19 (0.01) | 0.19 (0.01) | .75 | 0.53 (0.01) | 0.53 (0.01) | .88 | 0.15 (0.01) | 0.14 (0.004) | .62 | 0.32 (0.01) | 0.32 (0.01) | .60 |  |  |  |  |  |  |  |  |
| Hippocampus | 0.17 (0.01) | 0.20 (0.01) | .03* | 0.24 (0.02) | 0.28 (0.02) | .41 | 0.43 (0.01) | 0.44 (0.01) | 0.62 | 0.43 (0.01) | 0.45 (0.01) | 0.23 | 0.13 (0.01) | 0.11 (0.01) | 0.08 |  |  |  |  |  |  |  |  |
| Glu = glutamate; NAA = n-acetyl-aspartate; NAAG = n-acetyl aspartyl glutamate; mI = myoinositol; tCr = total creatine; Gln = glutamine, Glx = glutamate + glutamine; Cr = creatine; PCr = phosphocreatine; tCho = total choline; GSH = glutathione | | | | | | | | | | | | | | |  |  |  |  |  |  |  |  |  |

| Table S4. Significantly altered functional connectivity within resting state networks under the drug condition compared with placebo. | | | | | |
| --- | --- | --- | --- | --- | --- |
| Network | Cluster size | P_max_; | Effect size (mean beta) | MNI_max_ | Regions (N voxels; anatomical region) |
| Visual network 1 | 105 | 0.005 | -2.21 | 8, -78, -10 | 74 lingual gyrus r; 29 occipital fusiform gyrus r; 2 cerebellum 6 r |
| Visual  network 2 | 855 | <0.001 | -3.17 | -10, -84, 08 | 282 intracalcarine cortex l; 129 lingual gyrus l; 119 intracalcarine cortex r; 110 lingual gyrus r; 43 occipital pole l; 32 cuneal cortex l; 10 supracalcarine cortex l; 2 cuneal cortex r; 1 supracalcarine cortex r; 127 not labeled |
| Anterior DMN | 276  118  92 | <0.001  0.001  0,004 | -2.16  -2.00  -1,78 | -18, -48, 6  -8, 4, 22  -30, 26, -22 | Cluster 1: 134 precuneus cortex; 44 cingulate gyrus posterior division; 98 not labeled  Cluster 2: 118 atlas not labeled  Cluster 3:31 temporal fusiform cortex posterior division l; 19 hippocampus l; 12 parahippocampal gyrus posterior division l; 1 inferior temporal gyrus posterior division l; 1 parahippocampal gyrus anterior division l; 28 not labeled |
| Posterior DMN | 112 | 0.002 | -2.26 | -46, 22, -14 | 93 frontal orbital cortex l; 6 temporal pole l; 13 not labeled |
| Auditory network | 377  95 | <0.001  0.003 | -2.63  -2.52 | -62, -8, 4  -56, -36, 18 | Cluster 1: 64 planum temporale l; 59 central opercular cortex l; 53 superior temporal gyrus anterior division l; 23 superior temporal gyrus posterior division l; 22 planum polare l; 18 heschl;s gyrus l; 1 precentral gyrus l; 1 middle temporal gyrus anterior division l; 136 not labeled  Cluster 2: 39 parietal operculum cortex l; 39 planum temporale l; 5 supramarginal gyrus anterior division l; 12 not labeled |

| Table S5. Beta values from the between-network FC analysis, demonstrating increased functional connectivity between resting state networks under psilocybin compared to placebo. Asterisks indicate significant differences between treatment conditions (psilocybin > placebo, p < 0.05, FDR, two-tailed). | | | | | | | | | | | | | |
| --- | --- | --- | --- | --- | --- | --- | --- | --- | --- | --- | --- | --- | --- |
|  | Anterior DMN | Auditory | Cerebellum | Executive | Frontoparietal 1 | Frontoparietal 2 | Posterior DMN | Sensorimotor 1 | Sensorimotor 2 | Sensorimotor 3 | Visual 1 | Visual 2 | Visual 3 |
| Anterior DMN |  |  |  |  |  |  |  |  |  |  |  |  |  |
| Auditory | -0.03 |  |  |  |  |  |  |  |  |  |  |  |  |
| Cerebellum | 0.15* | 0.11 |  |  |  |  |  |  |  |  |  |  |  |
| Executive | 0.26* | 0.08 | 0.20* |  |  |  |  |  |  |  |  |  |  |
| Frontoparietal 1 | 0.20* | 0.04 | 0.10 | 0.11 |  |  |  |  |  |  |  |  |  |
| Frontoparietal 2 | 0.19* | 0.12 | 0.14* | 0.08 | 0.07 |  |  |  |  |  |  |  |  |
| Posterior DMN | -0.01 | 0.00 | 0.14* | 0.10 | 0.15* | 0.03 |  |  |  |  |  |  |  |
| Sensorimotor 1 | 0.00 | 0.19* | 0.03 | 0.16* | 0.09 | 0.12 | 0.05 |  |  |  |  |  |  |
| Sensorimotor 2 | -0.10 | -0.02 | 0.08 | 0.06 | 0.03 | 0.03 | 0.07 | 0.09 |  |  |  |  |  |
| Sensorimotor 3 | -0.07 | 0.17* | 0.05 | 0.06 | 0.06 | 0.13 | 0.02 | 0.20* | -0.01 |  |  |  |  |
| Visual 1 | 0.18* | 0.11 | 0.19* | 0.25* | 0.21* | 0.16* | 0.15* | -0.02 | 0.02 | 0.00 |  |  |  |
| Visual 2 | 0.19* | 0.09 | 0.27* | 0.21* | 0.20* | 0.18* | 0.11* | -0.01 | 0.05 | 0.01 | -0.18 |  |  |
| Visual 3 | 0.11 | 0.13* | 0.09 | 0.17* | 0.18* | 0.15* | 0.14* | 0.07 | 0.09 | 0.09 | -0.02 | -0.11 |  |

| Table S6. Overview of MRS data points that did not meet the data quality criteria check, and were subsequently not included in the analysis. Data points could be missing due to technological or participant difficulties. Once a spectra was acquired, quality was assessed and the entire spectra rejected if SNR< 10 and FWHM > 0.1. Finally, each metabolite was assessed individually, and discarded if relative CRLB > 20%. Psi = psilocybin. | | | | |
| --- | --- | --- | --- | --- |
|  | Full spectra | | | Per metabolite |
| Placement | Missing | SNR | FWHM | %CRLB |
| Medial prefrontal cortex | 7 (5 psi) | 0 | 0 | Glutamate: 0  GABA: 21 (10 psi)  NAA + NAAG: 0  Myoinositol: 0 |
| Hippocampus | 10 (7 psi) | 1 (0 psi) | 3 (2 psi) | Glutamate: 0  GABA: 33 (16 psi)  NAA + NAAG: 0  Myoinositol: 2 (1 psi) |

**Methods**

**Participants.** Participants were recruited through advertisements around Maastricht University and internet forum in the Netherlands. Inclusion criteria were: age, 18-40 years; previous experience with a psychedelic drug, but not within the past 3 months; normal weight, body mass index between 18 and 28 kg/m2; free from psychotropic medication; good physical health, including absence of major medical, endocrine, and neurological conditions; and written informed consent. Exclusion criteria were: history of drug abuse or addiction, which were determined by medical questionnaires and examination; pregnancy or lactation; health issues including hypertension (diastolic >90 and systolic >140), cardiac dysfunction, and liver dysfunction; current or history of psychiatric disorders; previous experience of serious side effects to cannabis; and MRI contraindications. Before inclusion, subjects were screened and examined by a study physician, who checked for general health, conducted a resting ECG, and took blood and urine samples in which hematology, clinical chemistry, urine, and virology analyses were conducted. Participant demographic data can be found in Table S1.

Psilocybin (powder) was obtained from GH Pharm GmbH, Frankfurt, Germany. A permit for obtaining, storing, and administering psilocybin was obtained from the Dutch Drug Enforcement Administration. Participants were financially compensated for their participation in the study.

**Procedure.** Participants were familiarized with the test day procedures on a separate training day prior to the treatment conditions. Participants were instructed to refrain from drug use, including psychedelic drugs (≥ 3 months), MDMA/ecstasy (≥ 14 days), alcohol (≥24 hours), and all other drugs of abuse (≥7 days) prior to their testing day. Additionally, participants were asked to refrain from caffeine and nicotine use the day of the test day.

On arrival of a test day, absence of drug and alcohol use was assessed via a urine drug screen and a breath alcohol screen. An additional pregnancy test was given if participants were female. If all tests were found to be negative, participants were allowed to proceed, and a venal catheter was placed, in order to take blood samples throughout the testing day. Before administration of treatment, a baseline blood sample was taken and baseline vital signs (blood pressure and heart rate) were measured. After measurements, the treatment was administered orally, in a closed cup containing bitter lemon (placebo) or bitter lemon and psilocybin (powder). After 40 minutes, participants were placed in the MRI scanner, where resting state scans and magnetic resonance spectroscopy were performed throughout a 1 hour time window. At the end of the test day (approximately 6 hours after treatment administration), participants were asked to complete measures of retrospective subjective high. Participants stayed under supervision until the testing day was complete, and the researcher deemed they were fit to go home.

**Questionnaires**

**5-Dimensional Altered States of Consciousness Rating Scale.** The 5D-ASC is a 94-item self-report scale that assesses the participants’ alterations from normal waking consciousness [^1^](#_ENREF_1). The participant is asked to make a vertical mark on the 10-cm line below each statement to rate to what extent the statements applied to their experience in retrospect from “No, not more than usually” to “Yes, more than usually.” The 5D-ASC contains the 5 key dimensions, including anxious ego dissolution, visual restructuralization, auditory alterations, reduction of vigilance, and oceanic boundlessness; which can be broken down into 11 subscales consisting of experience of unity, spiritual experience, blissful state, insightfulness, disembodiment, impaired control and cognition, anxiety, complex imagery, elementary imagery, audio-visual synesthesia, and changed meaning of percepts.

#### Ego Dissolution Inventory. The Ego Dissolution Inventory (EDI) is an eight-item self-report scale that assesses the participant’s experience of ego dissolution [^2^](#_ENREF_2). Sample items for the scale includes the following: “I experienced a dissolution of my ‘self’ or ego” and “I felt at one with the universe.” The participants answered the scale with endpoints of either 0 = “No, not more than usually” or 100 = “Yes I experience this completely/entirely.” The EDI is scored by calculating the mean of all the 8 items (range 0–100). The higher the total score, the stronger the experience of ego dissolution.

**Satisfaction with Life Scale.** The Satisfaction with Life Scale (SWLS) is a 5-item questionnaire designed to measure global cognitive judgments of satisfaction with one’s life [^3^](#_ENREF_3), and has been used to measure the life satisfaction component of subjective well-being [^3^](#_ENREF_3)^,^[^4^](#_ENREF_4). Individuals answer each item on a Likert-scale ranging from 1 (strongly disagree) to 7 (strongly agree). The total score is then obtained by summing the ratings from each item. Sample items include “In most ways my life is close to my ideal” and “If I could live my life over, I would change almost nothing”. The minimum possible score is 5, and a maximum possible score of 35, with a score of 5-19 defined as dissatisfied to below average life satisfaction, 20-24 defined as average life satisfaction, and 29-35 defined as high to very high life satisfaction [^5^](#_ENREF_5).

**Blood**

All samples were centrifuged and serum was frozen at -20°C, and kept in the dark until analysis. Analysis of psilocine in serum was performed according to [Martin, et al. ^6^](#_ENREF_6). Serum (200 µl) was extracted with 1 ml of ethyl acetate after addition of phosphate buffer pH 9, 20 ng psilocine-d_10_ and 10 µl of 0.1 M ascorbic acid for stabilization. The organic phase was evaporated and reconstituted with 100 µl of 0.1 % formic acid/acetonitrile (80:20, v/v). The analysis of 2 µl was performed on an Agilent (Waldbronn, Germany) LC-MS/MS system consisting of a 1290 Infinity Liquid Chromatograph coupled via JetStream Electrospray Interface (ESI) to a G6460A Triple Quadrupole Mass Spectrometer. Analytes were separated on a Kinetex® 2.6 µm XB-C18 100 Å LC column (100 x 2.1 mm) plus corresponding guard column from Phenomenex (Aschaffenburg, Germany) at 30 °C. Gradient elution at a flow rate of 0.5 ml/min using 0.01% formic acid containing 5 mM ammonium formate (A) and acetonitrile containing 0.1 % formic acid (B) started with 5 % B, increased to 95 % B during 4 min and was held for 2 min. Source parameters were: gas temperature 300 °C, gas flow 11 l/min, nebulizer 45 psi, sheath gas temperature 400 °C, sheath gas flow 12 l/min and capillary voltage 3500 V. Detection was performed in the multiple reaction monitoring mode (*m/z*, collision energy in parentheses, quantifier underlined): psilocine-d_10_: 215®66 (12), psilocine 205®58 (12); 205®160 (16). Five calibration standards were prepared from human serum with psilocine reference substance (LGC Standards GmbH, Wesel, Germany) and analyzed with the samples. The calibration was linear (regression coefficient >0.999) in the range 1 – 100 ng/ml with limits of detection and quantification below 0.5 ng/ml.

**MRS Acquisition.**

Anatomical (T_1_-weighted) images were acquired using magnetisation-prepared 2 rapid acquisition gradient-echo (MP2RAGE) [^7^](#_ENREF_7) sequence (TR = 4.5 s, TE = 2.39 ms, TI_1_ = 0.90 s, TI_2_ = 2.75 s, flip angle_1_ = 5°, flip angle_2_ = 3°, voxel size = 0.9 mm isotropic, matrix size = 256 × 256 × 192, phase partial Fourier = 6/8, GRAPPA factor = 3 with 24 reference lines, bandwidth = 250 Hz/pixel, acquisition time = 6:00 min). Tissue probability maps for grey matter (GM), white matter (WM) and cerebrospinal fluid (CSF) were generated from the T_1_-weighted anatomical images using FSL-FAST [^8^](#_ENREF_8), and assessed for differences in spectroscopic voxels, between groups.

Single-voxel proton magnetic resonance spectroscopy (MRS) measurements were performed on a MAGNETOM 7T MR scanner (Siemens Healthineers, Erlangen, Germany) with a whole-body gradient set (SC72; maximum amplitude, 70 mT/m; maximum slew rate, 200 T/m/s) and using an single-channel transmit/32-channel receive head coil (Nova Medical, Wilmington, MA, USA). Spectroscopic voxels of interest were placed by a trained operator at the medial prefrontal cortex (voxel size = 25 mm x 20 mm x 17 mm) and the right hippocampus (voxel size = 37 mm x 15 mm x 15 mm). Spectra were acquired with stimulated echo acquisition mode (STEAM) [^9^](#_ENREF_9) sequence using the following parameters: TE = 6.0 ms, TM = 10.0 ms, TR = 5.0 s, NA = 64, flip angle = 90°, RF bandwidth = 4.69 kHz, RF centred at 2.4 ppm, receive bandwidth = 4.0 kHz, vector size = 2048, 16-step phase cycling, acquisition time = 5:20 min. Water suppression was achieved by variable power RF pulses with optimised relaxation delays (VAPOR) [^10^](#_ENREF_10). In addition, a complete phase cycle of measurements was acquired without the water suppression RF pulses to record a water peak reference for eddy current correction [^11^](#_ENREF_11) and absolute metabolite concentration calibration [^12^](#_ENREF_12)^,^[^13^](#_ENREF_13). Before the spectroscopy measurements, a 3D-GRE dual-echo field-map (TE_1_ = 1.00 ms, TE_2_ = 2.98 ms, TR = 20.0 ms, flip angle = 8°, voxel size = 3 mm isotropic, matrix size = 84 × 84 × 56, bandwidth = 1450 Hz/pixel, acquisition time = 2:24 min) was acquired and used to calculate the shim currents required to homogenise the static magnetic field in the spectroscopic voxels of interest.

The spectra were analysed with LCModel version 6.3-1H using a GAMMA [^14^](#_ENREF_14) simulated basis set which includes Alanine (Ala), Ascorbic Acid (Asc), Aspartate (Asp), Creatine (Cr), γ-Aminobutyric Acid (GABA), Glucose (Glc), Glutamate (Glu), Glutamine (Gln), Glycerophosphocholine (GPC), Glutathione (GSH), Glycine (Glyc), Lactate (Lac), Myo-Inositol (mI), N-Acetyl Aspartate (NAA), N-Acetyl Aspartyl Glutamate (NAAG), Phosphocreatine (PCr), Phosphorylcholine (PCh), Phosphorylethanolamine (PE) , Scyllo-Inositol (Scyllo), and Taurine (Tau) [^15^](#_ENREF_15). The metabolite basis set also includes an in vivo Macromolecules (MMol) spectrum which was collected using a metabolites suppressed double inversion recovery (DIR) STEAM with the same parameters as above and TI_1_ = 2.09 s and TI_2_ =0.52 s [^16^](#_ENREF_16). All metabolite concentrations were reported with respect to total creatine. As metabolites were reported as a ratio to tCR, absolute metabolite concentration of tCR was checked in each brain region, for significant difference between groups. There was no significant difference between groups of tCR in either the mPFC or the hippocampus suggesting that any reported significant relative metabolite concentrations were not due to an increase or decrease in tCR

**MRS Quality**. To ensure data quality and reliable metabolite estimation, only absolute metabolite values with a relative Cramer–Rao lower bound below 20%, a signal-to-noise ratio (SNR) greater than 10, and a full-width at half-maximum peak height (FWHM) < 0.1 were considered[^17-19^](#_ENREF_17). MRS voxel placement and mean SNR, %CRLB, and FWHM values can be found in Table 1. An overview of data points that were *not* included in the analysis, and reason why, can be found in Table S6.

**Preprocessing.** Data was processed and analysed using the CONN toolbox 18.b[^20^](#_ENREF_20) (<http://www.nitrc.org/projects/conn)> based on SPM12 (<http://www.fil.ion.ucl.ac.uk/spm/)> running in MATLAB 2019a. Before quality assessement, FMRI data was available for 26 subjects in the psilocybin group and 27 subjects in the placebo group.

All functional volumes were realigned, unwarped, segmented into grey and white matter and cerebrospinal fluid, normalised into a standard stereotactic space (Montreal Neurological Institute; MNI) and smoothed with a 6 mm full width at half maximum Gaussian kernel. The first two volumes were excluded in order to to ensure magnetization equilibrium. No slice-time correction was performed as data was acquired using multi-band acquisition.

All individual T_1_-weighted structural volumes were segmented into grey and white matter and cerebrospinal fluid and normalised to MNI space. Noise correction of the functional images included scrubbing with a global signal threshold of z>3 and a composite subject motion threshold of >0.5 mm using ART as implemented in CONN, linear detrending, linear regression of the six motion parameters, and the white matter and cerebrospinal fluid signals, using the individual tissue masks obtained from the T_1_-weighted structural images. Five principal components were extracted from white matter and cerebrospinal fluid signals (using individual tissue masks obtained from the T1-weighted structural images) and removed using CompCor[^21^](#_ENREF_21). These components are thought to reflect noise (especially motion and physiological fluctuations) and are therefore removed from the time series. The resulting functional images were band-pass filtered (0.008 < f < 0.09 Hz) as it was found that band-pass filtering improves independent component results in addition to high-pass filtering (Pignat et al., 2013).

Quality assessment comprised three stages: Firstly, all scans were assessed with regard to the percentage of the scrubbed volumes. Subjects were only included in further analysis, if ≥ 5 min of the scan remained after scrubbing (corresponding to < 83.6% of the initial volumes). This was based on literature indicating that resting state scans < 5 min are not reliable (Birn et al., 2013). Secondly, head motion after scrubbing was assessed using maximum framewise displacement (FD; sphere radius 50 mm) calculated according to Power et al. (Power et al., 2012). Subjects were excluded if maximum FD was > 0.75 mm (half-voxel size). Four subjects (psilocybin group: 3; placebo group: 1) were excluded based on the first criterium and one subject (psilocybin group) was excluded based on the second criterium. The final sample thus consisted of 22 subjects in the psilocybin group and 26 subjects in the placebo group. All further analyses were based on this sample. Furthermore, we tested for significant differences between the groups of this sample. Mean FD before scrubbing was 0.18 mm (± SD 0.05) in the psilocybin group and 0.18 mm (± SD 0.06) in the placebo group. Average maximum FD before scrubbing in the psilocybin group was 0.98 mm (± SD 0.78) in the psilocybin group and 0.99 mm (± SD 0.95) in the placebo group. On average 10.90 volumes per scan (± SD 9.49) were scrubbed in the psilocybin group and 10.50 volumes per scan (± SD 9.80) in the placebo group. Mean FD after scrubbing was 0.17 mm (± SD 0.05) in the psilocybin group and 0.17 mm (± SD 0.05) in the placebo group. Average maximum FD before scrubbing in the psilocybin group was 0.51 mm (± SD 0.12) and 0.50 mm (± SD 0.13) in the placebo group. For comparison between groups, Mann-Whitney U tests were performed because the assumption for normality distribution was not met in several cases (assessed with Shapiro-Wilk tests). There were no significant differences between groups for any of these measures (invalid volumes: p=0.77; mean FD before scrubbing: p=0.72; average maximum FD before scrubbing: p=0.81; mean FD after scrubbing: p=0.81; average maximum FD after scrubbing: p=0.70).

**Independent component analysis**. Independent component analysis (ICA) was performed using group-ICA procedures implemented in the CONN toolbox following methods described by Calhoun et at. (Calhoun et al., 2001). ICA results are determined by the chosen number of dimensions, i.e. a higher number of dimensions might result in a higher number of distinct resting state networks compared with a lower number of dimensions. Dimensionality reduction on the subject-level was set to 64. Independent components were restricted to 20 in order to allow comparisons with 10 established resting state network described by Smith et al. (Smith et al., 2009) and previous studies on psilocybin (Carhart-Harris et al., 2013) and LSD (Carhart-Harris et al., 2016; Müller et al., 2018), which also applied comparable restrictions. Decisions regarding the labelling of the networks identified in this data set were based on visual inspection (Kelly  Jr. et al., 2010) and cross-correlation of the unthresholded ICA components with the unthresholded resting state networks described by Smith et al. (<https://www.fmrib.ox.ac.uk/datasets/brainmap+rsns/>).

**References**

1 Studerus, E., Gamma, A. & Vollenweider, F. X. Psychometric evaluation of the altered states of consciousness rating scale (OAV). *PLoS ONE* **5**, e12412 (2010).

2 Nour, M. M., Evans, L., Nutt, D. & Carhart-Harris, R. L. Ego-dissolution and psychedelics: validation of the ego-dissolution inventory (EDI). *Frontiers in human neuroscience* **10**, 269 (2016).

3 Diener, E., Emmons, R. A., Larsen, R. J. & Griffin, S. The Satisfaction With Life Scale. *Journal of personality assessment* **49**, 71-75, doi:10.1207/s15327752jpa4901_13 (1985).

4 Pavot, W., Diener, E., Colvin, C. R. & Sandvik, E. Further validation of the Satisfaction with Life Scale: evidence for the cross-method convergence of well-being measures. *Journal of personality assessment* **57**, 149-161, doi:10.1207/s15327752jpa5701_17 (1991).

5 Pavot, W. & Diener, E. *The Satisfaction with Life Scale (SWL). Measurement Instrument Database for the Social Science*, <[www.midss.ie](http://www.midss.ie)> (2013).

6 Martin, R., Schurenkamp, J., Pfeiffer, H. & Kohler, H. A validated method for quantitation of psilocin in plasma by LC-MS/MS and study of stability. *International journal of legal medicine* **126**, 845-849, doi:10.1007/s00414-011-0652-8 (2012).

7 Marques, J. P. *et al.* MP2RAGE, a self bias-field corrected sequence for improved segmentation and T1-mapping at high field. *NeuroImage* **49**, 1271-1281, doi:10.1016/j.neuroimage.2009.10.002 (2010).

8 Zhang, Y., Brady, M. & Smith, S. Segmentation of brain MR images through a hidden Markov random field model and the expectation-maximization algorithm. *IEEE transactions on medical imaging* **20**, 45-57, doi:10.1109/42.906424 (2001).

9 Frahm, J. *et al.* Localized high-resolution proton NMR spectroscopy using stimulated echoes: initial applications to human brain in vivo. *Magnetic resonance in medicine* **9**, 79-93 (1989).

10 Tkac, I., Starcuk, Z., Choi, I. Y. & Gruetter, R. In vivo 1H NMR spectroscopy of rat brain at 1 ms echo time. *Magnetic resonance in medicine* **41**, 649-656 (1999).

11 Klose, U. In vivo proton spectroscopy in presence of eddy currents. *Magnetic resonance in medicine* **14**, 26-30 (1990).

12 Barker, P. B. *et al.* Quantitation of proton NMR spectra of the human brain using tissue water as an internal concentration reference. *NMR in biomedicine* **6**, 89-94 (1993).

13 Soher, B. J., Hurd, R. E., Sailasuta, N. & Barker, P. B. Quantitation of automated single-voxel proton MRS using cerebral water as an internal reference. *Magnetic resonance in medicine* **36**, 335-339 (1996).

14 Smith, S. A., Levante, T. O., Meier, B. H. & Ernst, R. R. Computer Simulations in Magnetic Resonance. An Object-Oriented Programming Approach. *Journal of Magnetic Resonance* **106**, 75-105 (1994).

15 Govindaraju, V., Young, K. & Maudsley, A. A. Proton NMR chemical shifts and coupling constants for brain metabolites. *NMR in biomedicine* **13**, 129-153 (2000).

16 Penner, J. & Bartha, R. Semi-LASER 1 H MR spectroscopy at 7 Tesla in human brain: Metabolite quantification incorporating subject-specific macromolecule removal. *Magnetic resonance in medicine*, doi:10.1002/mrm.25380 (2014).

17 Provencher, S. W. Automatic quantitation of localized in vivo 1H spectra with LCModel. *NMR in biomedicine* **14**, 260-264 (2001).

18 Kreis, R. Issues of spectral quality in clinical 1H-magnetic resonance spectroscopy and a gallery of artifacts. *NMR in biomedicine* **17**, 361-381, doi:10.1002/nbm.891 (2004).

19 Wilson, M. *et al.* Methodological consensus on clinical proton MRS of the brain: Review and recommendations. *Magnetic resonance in medicine* **82**, 527-550, doi:10.1002/mrm.27742 (2019).

20 Whitfield-Gabrieli, S. & Nieto-Castanon, A. Conn: a functional connectivity toolbox for correlated and anticorrelated brain networks. *Brain connectivity* **2**, 125-141, doi:10.1089/brain.2012.0073 (2012).

21 Behzadi, Y., Restom, K., Liau, J. & Liu, T. T. A component based noise correction method (CompCor) for BOLD and perfusion based fMRI. *NeuroImage* **37**, 90-101, doi:10.1016/j.neuroimage.2007.04.042 (2007).
